# Concurrence of Crossed Cerebellar Diaschisis and Parakinesia Brachialis Oscitans in a Patient with Hemorrhagic Stroke

**DOI:** 10.1155/2013/519808

**Published:** 2013-11-06

**Authors:** Yung-Tsan Wu, Shin-Tsu Chang, Liang-Cheng Chen, Tsung-Ying Li

**Affiliations:** ^1^Department of Physical Medicine and Rehabilitation, Tri-Service General Hospital, School of Medicine, National Defense Medical Center, No. 325, Sec. 2, Cheng-Kung Road, Neihu District, Taipei 114, Taiwan; ^2^Department of Rehabilitation, Taichung Veterans General Hospital, Taichung 407, Taiwan

## Abstract

Crossed cerebellar diaschisis (CCD) is defined as a reduction in blood flow in the cerebellar hemisphere contralateral to the supratentorial focal lesion. The phenomenon termed parakinesia brachialis oscitans (PBO) in which stroke patients experience involuntary stretching of the hemiplegic arm during yawning is rarely reported. The concurrence of CCD and PBO has never been described. A 52-year-old man had putaminal hemorrhage and demonstrated no significant recovery in his left hemiplegia after intensive rehabilitation, but his gait improved gradually. Two months after the stroke, the single photon emission computed tomography (SPECT) showed CCD. Four months after the stroke, the patient noticed PBO. The follow-up SPECT showed persistent CCD and the patient's arm was still plegic. The frequency and intensity of PBO have increased with time since the stroke. We speculate that the two phenomena CCD and PBO might share similar neuroanatomical pathways and be valuable for predicting clinical recovery after stroke.

## 1. Introduction

Crossed cerebellar diaschisis (CCD) is defined as a reduction in blood flow in the cerebellar hemisphere contralateral to a supratentorial focal lesion such as a cerebral infarction or hematoma. CCD occurs in more than 50% of patients with supratentorial focal lesions. Relatively few articles have described CCD due to a subcortical hemorrhage [1]. In addition, less attention has been paid to the phenomenon termed parakinesia brachialis oscitans (PBO) in which stroke patients experience involuntary stretching of the hemiplegic arm upon yawning [2]. The anatomical pathways involved in this involuntary motor response are yet to be fully clarified, and the prevalence of PBO is rarely reported.

The concurrence of PBO and CCD has never been described. Here, we report the case of a stroke patient with putaminal hemorrhage presenting with concurrent CCD and PBO and discuss their common anatomic pathway and the effects of these conditions on clinical outcome.

## 2. Case Report

A 52-year-old right-handed man, who suffered from hypertension for 5 years without regular medicine and followup, was admitted to our facility after acute loss of consciousness. The patient exhibited left hemiplegia secondary to right putaminal hemorrhage (3 × 5 cm in size) extending into the posterior portion of the internal capsule with the mass effect. On neurological examination, the patient had weak muscle power (1/5 in the left upper limb and 2/5 in the left lower limb) and positive Babinski sign on the left side. An emergent craniotomy for removal of a hematoma was performed; however, the patient demonstrated no significant recovery in his left hemiplegia after the operation. Results of a Romberg test and dynamic testing were poor. The patient's Functional Independence Measurement (FIM) score was 50, and his Barthel index (BI) was 25. Amlodipine besylate 5 mg qd, valsartan 80 mg qd, and atorvastatin 10 mg qd were prescribed for secondary prevention of cerebrovascular disease. Color Doppler sonography showed only mild degree of intimal thickening and sparse mural calcified plaques along the course of bilateral extracranial common and internal carotid and vertebral arteries as well without significant stenosis. MR angiography of the circle of Willis shows decreased signal intensity and number of branches of right middle cerebral artery.

**Figure 1:**
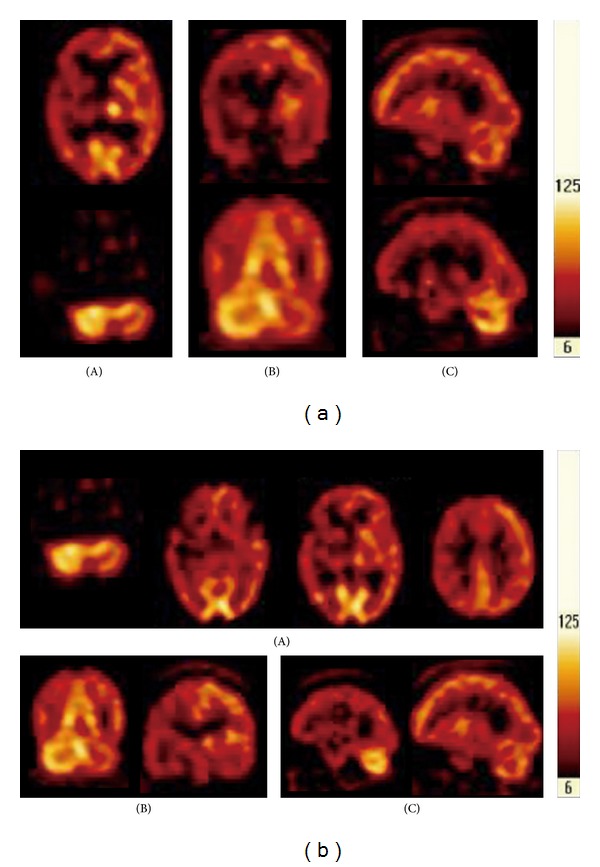
(a) The pictures of the brain SPECT. (A) Transverse view; (B) coronal view; (C) sagittal view. There is inhomogeneous perfusion in the cerebral cortex with relative decrease of uptake in right fronto-temporal-parietal, right temporal, and right parietal regions. The uptake in right basal ganglion and thalamus is decreased when compared with the left side. Remarkably, the uptake in left cerebellum looks lower than the right cerebellum, suggesting the presence of CCD. (b) The second examination of the brain SPECT. (A) Transverse view; (B) coronal view; (C) sagittal view. The pictures checked in the second time are similar to the first, and interestingly enough, CCD remains in the left cerebellum.

After intensive rehabilitation, the patient's hemiplegia remained, but his gait improved gradually. Two months after the stroke, muscle power was reevaluated in the left upper and lower limbs and was 2/5 and 3/5, respectively. Single photon emission computed tomography (SPECT) showed decreased perfusion in the right cerebral cortex and in the left cerebellum. CCD on the left side was confirmed (Figure 1(a)). Four months after the stroke, the patient noticed that he involuntarily stretched his hemiplegic arm when yawning in bed. The movement consisted of a progressive abduction, anteroflexion, and mild internal rotation of the shoulder, followed by arm lifting with a flexion of the elbow. The movement lasted for a few seconds. The involuntary activity was absent when the patient was in a sitting position. Repeated attempts to induce yawning by imitation were unsuccessful.

Eight months after the stroke, a follow-up SPECT showed persistent CCD (Figure 1(b)). The patient's arm was still plegic, but he could walk slowly using a cane with the assistance of an ankle-foot orthosis. The follow-up FIM score and BI were 102 and 70, respectively. The patient continues to experience persistent hemiplegia and CCD, and his functional index has improved only gradually. The frequency and intensity of the phenomenon increased steadily with time after the stroke and also occured when sitting.

## 3. Discussion

To the best of our knowledge, the present case with putaminal hemorrhage together with CCD and PBO is the first such case to be reported. Relatively few studies have been published on CCD after intracerebral hemorrhage; however, similar studies on cerebral infarction are reported. The SPECT is a nuclear medicine tomographic imaging technique and physicians often utilize it for functional brain imaging to assess blood perfusion of the brain such as CCD. With respect to the relationship between prognosis and CCD, Sobesky et al. [3] reported that the CCD was significantly corrected with the degree of supratentorial hypoperfusion and persistent CCD closely linked to poor clinical outcome and permanent supratentorial tissue damage. Szilágyi et al. [4] also revealed that the severity of CCD could be with a quantitative predictor of functional impairment in stroke patients. In contrast, Flint et al. [5] disagreed the relationship between severity of CCD and volume of ischemic stroke.

The neuroanatomical pathways contributing to CCD have been described as being activated by disruption of the corticopontocerebellar tract [6], which mainly connects with the corticospinal tract in the posterior limb of the internal capsule. The corticopontocerebellar fibers are in close contact with the corticospinal tract, but are more extensive than the corticospinal fibers. In previous studies, most cases with CCD showed pyramidal tract dysfunction. A reasonable explanation could be that these two fibers are close to each other and that most stroke patients have pyramidal tract dysfunction. Pantano et al. [7] also suggested that destruction of the pyramidal tract is not necessary for occurrence of CCD since some patients without hemiparesis have CCD, while not all patients with hemiparesis have CCD.

**Figure 2:**
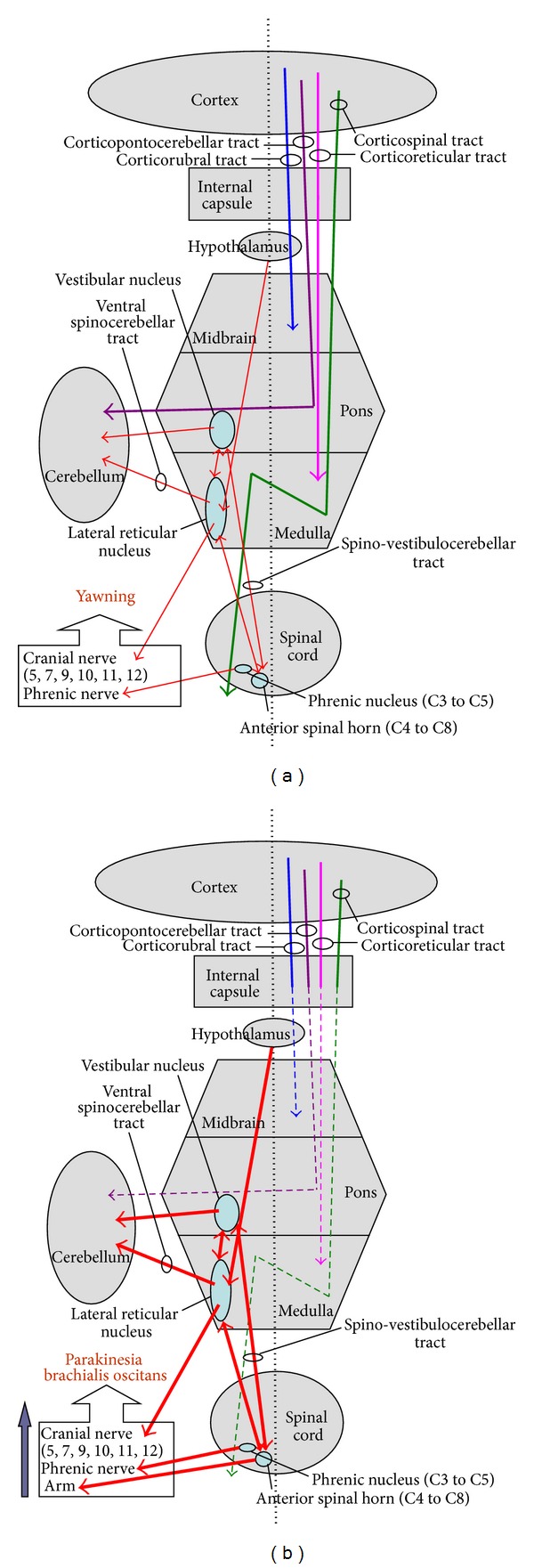
(a) Schematic representation of neuroanatomical pathways ofyawning system. (b) Schematic representation of neuroanatomical pathways of crossed cerebellar diaschisis and parakinesia brachialis oscitans.

The phenomenon of PBO described as unintentional hemiplegia-associated movement during yawing may appear during the flaccid or spastic phase and tended to disappear when neurological recovery was noted [2, 8]. The variants of classic PBO in two case reports without paretic upper extremity were described by de Lima et al. [9]. The yawning center has not been truly identified. Some clinical evidence suggests the major areas are pons, medulla, basal ganglion, and hypothalamus, particularly the paraventricular nucleus (PVN). The PVN project to the lateral reticular formation (couple ventilation and locomotion in animals) and locus ceruleus of brainstem to trigger yawning by exciting the cranial nerve (V, VII, IX, X, XI, and XII) and phrenic nerve (Figure 2(a)) [10].

Previous studies have concluded that most PBO patients had lesions in the posterior limb of internal capsule, involving damage to the first neuron and interrupting the corticospinal, corticonuclear, corticorubral, corticostriate, corticonigral, and corticoreticular pathways [2, 11]. Walusinski et al. [2, 8] found that the corticoneocerebellospinal pathway (such as corticopontocerebellar tract) is interrupted in certain PBO cases, but the conducting system of proprioception between the motor anterior spinal horn, the paleocerebellum, and the lateral reticular nucleus (spinoreticulocerebellar tract) remains intact. They also speculated that the mechanism of involuntary stretching of the hemiplegic arm upon yawning is a motor signal of anterior spinal horn in C4 to C8 which originate in the lateral reticular nucleus and travel through the extrapyramidal pathways of the archeocerebellum (such as spinovestibulocerebellar tract). Moreover, they suggest that interruption of corticonuclear, corticospinal, and corticoneocerebellar disinhibiting the spinoarcheocerebellar tract might be the major mechanism of PBO (Figure 2(b)).

According to the known and hypothesized mechanisms mentioned above, CCD and PBO seem to share similar neuroanatomical pathways such as corticopontocerebellar tract and the posterior limb of the internal capsule. The possible basis of the concurrence of CCD and PBO in our patient was the putaminal hemorrhage that extended to the posterior limb of the internal capsule.

The involuntary stretching in the plegic arm might be useful in promoting muscular strengthening of the affected limb. We believe that if this yawning behavior could be stimulated by medication or another mechanism, the plegic performance of our patient might improve. However, in our case, exploration of this possibility was limited, and we could arrive at no conclusion with regard to this issue. Further studies will be carried out in the future.

Whether the persistence of CCD and PBO in this patient corresponded to the poor recovery of left hemiplegia is uncertain because no similar case has been reported. The prognosis in putaminal hemorrhage is related to many factors such as Glasgow Coma Scale score, pyramidal sign to the nonhemiplegic side, midline shift, size of hematoma, and cerebellar perfusion. We speculate that the two phenomena CCD and PBO may be valuable for predicting clinical recovery after stroke when they occur together.
